# Polymorphisms in *GCKR*, *SLC17A1* and *SLC22A12* were associated with phenotype gout in Han Chinese males: a case–control study

**DOI:** 10.1186/s12881-015-0208-8

**Published:** 2015-08-20

**Authors:** Zhao-Wei Zhou, Ling-Ling Cui, Lin Han, Can Wang, Zhi-Jian Song, Jia-Wei Shen, Zhi-Qiang Li, Jian-Hua Chen, Zu-Jia Wen, Xiao-Min Wang, Yong-Yong Shi, Chang-Gui Li

**Affiliations:** Shandong Gout Clinical Medical Center, The Affiliated Hospital of Qingdao University, 16 Jiangsu Road, Qingdao, 266003 China; Shandong Provincial Key Laboratory of Metabolic Disease, The Affiliated Hospital of Qingdao University, 16 Jiangsu Road, Qingdao, 266003 China; Bio-X Institutes, Key Laboratory for the Genetics of Developmental and Neuropsychiatric Disorders (Ministry of Education), Shanghai Jiao Tong University, Shanghai, 200030 China

**Keywords:** Gout, SNPs, *GCKR*, *SLC17A1*, *SLC22A12*

## Abstract

**Background:**

Gout is a common arthritic disease resulting from elevated serum uric acid (SUA) level. A large meta-analysis including 28,141 individuals identified nine single nucleotide polymorphisms (SNPs) associated with altered SUA level in a Caucasian population. However, raised SUA level alone is not sufficient for the development of gout arthritis and most of these SNPs have not been studied in a Han Chinese population. Here, we performed a case–control association analysis to investigate the relationship between these SUA correlated SNPs and gout arthritis in Han Chinese.

**Methods:**

A total of 622 ascertained gout p9atients and 917 healthy controls were genotyped. Genome-wide significant SNPs, rs12129861, rs780094, rs734553, rs742132, rs1183201, rs12356193, rs17300741 and rs505802 in the previous SUA study, were selected for our analysis.

**Results:**

No deviation from the Hardy–Weinberg equilibrium was observed either in the case or control cohorts (corrected p > 0.05). Three SNPs, rs780094 (located in *GCKR*, corrected p = 1.78E^−4^, OR = 0.723), rs1183201 (located in *SLC17A1*, corrected p = 1.39E^−7^, OR = 0.572) and rs505802 (located in *SLC22A12*, corrected p = 0.007, OR = 0.747), were significantly associated with gout on allelic level independent of potential cofounding traits. While the remaining SNPs were not replicated. We also found significant associations of uric acid concentrations with these three SNPs (rs780094 in *GCKR*, corrected p = 3.94E^−5^; rs1183201 in *SLC17A1,* corrected p = 0.005; rs505802 in *SLC22A12*, corrected p = 0.003) and of triglycerides with rs780094 (located in *GCKR,* corrected p = 2.96E^−4^). Unfortunately, SNP-SNP interactions for these three significant SNPs were not detected (rs780094 vs rs1183201, p = 0.402; rs780094 vs rs505802, p = 0.434; rs1183201 vs rs505802, p = 0.143).

**Conclusions:**

Three SUA correlated SNPs in Caucasian population, rs780094 in *GCKR,* rs1183201 in *SLC17A1* and rs505802 in *SLC22A12* were confirmed to be associated with gout arthritis and uric acid concentrations in Han Chinese males. Considering genetic differences among populations and complicated pathogenesis of gout arthritis, more validating tests in independent populations and relevant functional experiments are suggested in future.

## Background

Gout is the most common cause of arthritis and elevated serum uric acid (SUA) level, namely hyperuricemia, is an important independent risk factor for gout [1]. There are extensive data which suggests that the incidence and prevalence of hyperuricemia and gout increased markedly over the past decades worldwide [2–5]. In addition, hyperuricemia and gout have been independently linked to other endocrine and metabolic diseases including obesity, insulin resistance, diabetes, dyslipidemia and cardiovascular diseases like hypertension and coronary heart disease [1]. Gout is a lifelong disease inflicting a considerable burden of illness upon employers in terms of treatment costs as well as other work-related “benefits” [6].

In addition to known environmental factors such as high-purine diet, drinking and smoking [1], genetic components are also involved in the occurrence of hyperuricemia and gout, with heritability up to 40 % [7]. Identifying genetic factors is necessary to improve the etiological diagnosis and management of hyperuricemia and gout. With the rapid development of genetic methods, especially genome-wide association studies (GWAS), dozens of variants of genes that are associated with SUA levels or/and gout are found now. And most of these genes are involved with the renal uric acid-transport system rather than inflammatory pathways. Among these, the most significant findings are variants located within *ABCG2* (ATP-binding cassette, sub-family G, member 2), *SLC2A9* (solute carrier family 2 member 9) and *SLC22A12* (solute carrier family 22 member 12), having been consistently replicated across different ethnic groups including Europeans, Chinese, Japanese, Koreans and Mexican Americans [8–16]. Kolz et al. performed a large meta-analysis including 28,141 individuals and identified nine different loci associated with SUA levels. These included the previously reported genes *SLC22A12*, *SLC2A9*, *ABCG2*, *SLC17A1-SLC17A3* and the newly identified genes *PDZK1*, *GCKR*, *LRRC16A*, *SLC16A9* and *SLC22A11* [17]. Another large-scale GWAS from >140,000 individuals of European ancestry for SUA level also replicated most of these nine significant loci and additional 18 new loci [18]. A study of Netherland residents corroborated the association of *PDZK1*, *GCKR*, *SLC16A9* and *SLC22A11* loci with altered SUA concentrations, but failed to replicate the *LRRC16A* locus as a risk factor [19]. A study of Germans investigated the association of all the above SNPs with gout and only identified rs734553 in *SLC2A9* and rs2231142 in *ABCG2* to be risk factors [20]. The “A” allele of rs742132 in the *LRRC16A* gene was found to be risk allele for gout in two independent association studies both from Japanese origin [21, 22]. The “A” allele of rs780094 in *GCKR* gene has been validated to be risk allele for gout consistently in Chinese, Japanese and New Zealand European and Polynesian case–control sample sets [22–24]. SNP rs1183201 in *SLC17A1* was also corroborated to be associated with gout in New Zealand Caucasians [24, 25] and rs1165205 in *SLC17A3* (in high linkage disequilibrium (LD) with rs1183201 in HapMap-CEU, r^2^ = 0.889) was replicated in European whites [8]. Noticeably, a recent GWAS in Chinese population identified two previously reported SUA loci of *SLC2A9* (rs11722228) and *ABCG2* (rs2231142, rs4148152 and rs3114018, rs4148155), but failed to replicate the remaining loci which were associated with SUA level in Europeans [11]. Other replication studies from Han Chinese origin also reported additional associations for variants in these loci with gout disease, especially for *SLC2A9* [25–27], *ABCG2* [28–31]and *SLC22A12* [32–34]. LD structure between these variants may differ among different ethnics. Both rs16890979 and rs6855911 were in strong LD with rs734553 in HapMap-CEU (r^2^ = 0.957 and 1.0, respectively) and were confirmed to be associated with gout in Europeans [8, 20]. In Chinese population, rs6855911 (in strong LD with rs734553 in HapMap-CHB, r^2^ = 1.0) was not identified in gout-control groups but was replicated in high-uric-acid and normal-uric-acid groups [26] and rs16890979 was in much lower LD with rs734553 from HapMap-CHB (r^2^ = 0.494). *GCKR* rs780093 (in strong LD with rs780094 in HapMap-CEU and HapMap-CHB, r^2^ = 1.0) was nominally associated with gout in Europeans and Chinese [12, 23]. *SLC17A1* rs1165196 (in strong LD with rs1183201 from HapMap-CEU, r^2^ = 0.889) was associated with SUA level in whites [8, 12]. However, both rs1165196 and 1183201 (in strong LD with r^2^ = 0.904 in HapMap-CHB) showed no significant difference between the case–control groups [35]. Besides, another GWAS and meta-analysis both for SUA level in Japanese population also corroborated the three well-known loci of *SLC2A9* (rs11722228 and rs3775948), *ABCG2* (rs4148155 and rs2725220) and *SLC22A12* (rs506338 and rs504915) [13, 14]. Considering the inconsistency among these association studies and different genetic structure in different populations, a further validation study in different populations is necessary. Therefore, we performed a genetic association study of these SUA loci (*PDZK1, GCKR, SLC2A9, LRRC16A, SLC17A1, SLC16A9, SLC22A11* and *SLC22A12*) in gout patients and normal control volunteers of Han Chinese origin. SNP rs2231142 in *ABCG2* were excluded from this study since our group and other domestic institutions have clearly clarified consistent association between this site and gout [27–29, 31, 35]. Besides, we don’t assess the relationship of the 18 new loci with gout in the present study considering that the proportion of variance in SUA concentrations explained by these new loci was as low as 1.8 % in total [18].

## Methods

### Participants and Phenotypes

All the patients and controls were of Han Chinese origin and had long-term residence in the coastal areas of Shandong Province. A total of 622 unrelated cases were recruited from the gout clinic at the Affiliated Hospital of Qingdao University and were diagnosed with primary gout by experienced physicians according to criteria established by the American College of Rheumatology [36]. All 917 unrelated controls who had SUA values below 420 μmol/L, and never suffered from an acute attack of gouty arthritis were recruited. All participants with relevant medication including allopurinol and lipid-lowering drugs etc., a family history of gout and severe illness, such as hepatitis or cancer, were excluded. Notably, only males were recruited in this study due to less than 5 % of our outpatients were female, not sufficient to conduct a statistical analysis. This study was approved by the Ethics Committee of Affiliated Hospital of Qingdao University. All participants gave their written informed consent. The study was in accordance with the principles of the current version of the Declaration of Helsinki.

Phenotype details including age, height and weight were collected in a questionnaire at the time of admission and body mass index (BMI) was calculated from the calculation formula weight (kg)/height (m) ^2^. Systolic blood pressure (mmHg) and diastolic blood pressure (mmHg) were measured and recorded by physicians on our gout clinic. Related biochemical indicators including blood glucose, triglycerides, total cholesterol, urea nitrogen, creatinine and uric acid in the plasma of all the participants were measured using an automated multichannel chemistry analyzer (Model 200; Toshiba, Tokyo, Japan). The information of phenotype details and biochemical indicators in the two cohorts is listed in Table 1.

**Table 1:** Characteristics of gout patients and controls

Variable	Cases (*N* = 622)	Controls (*N* = 917)	*P* value
Age (year)	51.95 ± 13.68	47.56 ± 12.70	<0.001
BMI (kg/m^2^)	27.23 ± 3.77	25.401 ± 3.10	<0.001
SBP (mmHg)	134.07 ± 17.06	132.31 ± 19.53	0.098
DBP (mmHg)	87.75 ± 12.19	84.15 ± 12.19	<0.001
Glu (mmol/L)	6.50 ± 1.76	5.87 ± 1.85	<0.001
Tri (mmol/L)	2.49 ± 2.25	1.53 ± 1.17	<0.001
Cho (mmol/L)	5.15 ± 1.13	5.31 ± 1.43	0.025
UN (mmol/L)	5.34 ± 2.50	5.39 ± 2.42	0.715
Cre (μmol/L)	88.00 ± 40.42	80.34 ± 17.54	<0.001
UA (μmol/L)	475.88 ± 123.04	297.97 ± 47.41	<0.001

Values denote means ± standard deviations; *BMI*, body mass index; *SBP*, Systolic blood pressure; *DBP*, Diastolic blood pressure; *Glu*, Blood glucose; *Tri*, Triglycerides; *Cho*, Cholesterol; *UN*, Urea nitrogen; *Cre*, Creatinine; *UA*, Uric acid

### SNP Selection and Genetic Analyses

Eight SNPs (rs12129861 at Chr1, rs780094 at Chr2, rs734553 at Chr4, rs742132 at Chr6, rs1183201 at Chr6, rs12356193 at Chr10, rs17300741 at Chr11, rs505802 at Chr11) were determined by a large-scale meta-analysis for SUA values (shown in Table 2). SNP rs2231142 in *ABCG2* were excluded from this study since our group and other domestic institutions have clearly clarified consistent association between this site and gout [27–29, 31, 35]. Genomic DNA was extracted from peripheral leukocytes according to the manufacturer’s protocols (Lifefeng Biotech Co., Ltd, Shanghai, China). Extracted DNA was confirmed and quantified with a NanoDrop 1000 Spectrophotometer (Thermo Scientific, USA). For the genotyping of these SNPs, PCR amplification was performed using the Gene Amp PCR System 9600 (Applied Biosystems, Foster City, CA, USA). 3 % agarose gel electrophoresis was performed to separate the PCR products. Finally, DNA genotyping was performed using PRISM 3730 instruments (Applied Biosystems, Foster City, CA, USA). The primer sequences (included as additional files) were designed using Primer 3 online Version 0.4.0 and obtained from Hanyu Biotech Co., Ltd, Shanghai, China.

**Table 2:** Summary of eight SNPs used in analysis

SNP	Position^a^	Gene name and function	Minor allele^b^	Populations and reference	MAF^c^ CEU CHB
rs12129861	1q21.1	*PDZK1*, 5’Intergenic	A	European (17, 19, 20)	0.460 0.170
rs780094	2p23.3	*GCKR*, Intron16	A	European (17, 19, 20)	0.394 0.566
rs734553	4p10.1	*SLC2A9*, Intron7	C	European (17, 19, 20)	0.261 0.004
rs742132	6p22.2	*LRRC16A*, Intron34	C	European (17, 20)	0.301 0.244
				Japanese (21)	
rs1183201	6p22.2	*SLC17A1*, Intron 3	A	European (17,20)	--- ---
rs12356193	10q21.2	*SLC16A9*, Intron 5	G	European (17, 19, 20)	0.186 0.141
rs17300741	11q13.1	*SLC22A11*, Intron4	G	European (17, 19, 20)	0.531 0.073
rs505802	11q13.1	*SLC22A12*, 5’Intergenic	A	European (17, 19, 20)	0.726 0.256

^a^on human genome build 18

^b^in NCBI

^c^collected from HapMap Data Phase III/Rel#3. CEU: Utah residents with Northern and Western European ancestry from the CEPH collection, CHB: Han Chinese in Beijing, China

### Statistical Analyses

Clinical data between case–control cohorts was compared using a t test by SPSS 19.0. Before the association analysis was performed, Hardy-Weinberg equilibrium (HWE) for the case and control group respectively was evaluated to determine whether these genotype distributions achieved genetic equilibrium (threshold set to 0.05). In the case–control study, the differences in allele frequencies were compared using chi-square test with odds ratios (ORs) and their 95 % confidence intervals (CI) reported. Logistic regression which considered each of the potential confounding covariates was performed to indicate that gout was or wasn’t the major contributor to the genetic associations. An analysis of variables was conducted to study the differences between genotypes and clinical characteristics in all participants. False Discovery Rate (FDR) correction was applied at the same time due to multiple comparisons. After correction, a p-value less than 0.05 was considered statistically significant. All these association analyses were performed using the online software SHEsis (http://shesisplus.bio-x.cn/) [37], a powerful and user-friendly software platform for genetic association analysis.

SNP-SNP interactions were conducted using a logistic regression analysis with the second SNP as a covariate also by SHEsis (http://shesisplus.bio-x.cn/) [37]. Power was calculated using obtained unadjusted OR and probability of exposure in controls with preestablished α error probability (α = 0.05 for a two sided test) using Power and Sample Size Calculation Software [38].

## Results

### Phenotype details and biochemical indicators

T test results for phenotypic characteristics between the two independent cohorts are listed in Table 1. The age of the two groups was significantly different (51.95 ± 13.68 vs 47.56 ± 12.70 years, p < 0.001). Body mass index (BMI), diastolic blood pressure and blood glucose, triglycerides, creatinine and uric acid levels of gout patients were significantly higher than those of controls (all *p* < 0.001). Cholesterol levels of gout patients were lower than those of controls (*p* = 0.025). Systolic blood pressure and urea nitrogen level of the two groups were not statistically different (*p* = 0.098 and *p* = 0.715, respectively).

### SNP association with gout risk

All subjects were genotyped for the eight SNPs and all SNPs obtained 97.9 % call rates in genotyping (data not shown in the tables). Up to 18 internal positive and negative duplicates were examined to control the genotyping quality and genotyping results were consistent.

All SNPs achieved genetic equilibrium in both case and control cohorts (HWE corrected p > 0.05; shown in Table 3).

**Table 3:** Association analysis of 8 SUA-related SNPs with gout in Chinese Han males

SNP	HWE^a^	Effect	Frequency	Allele	Allelic OR	FDR	Power
		allele^b^	(case, ctrl)	p-value	% 95 CI		
rs12129861	0.999	A	0.163	0.327	0.907	1	11.4 %
	0.393		0.177		[0.747 ~ 1.101]		
rs780094	0.999	G	0.394	1.64E^−5^	0.723	**1.78E** ^**−4**^	87.6 %
	0.997		0.473		[0.624 ~ 0.838]		
rs734553	0.999	C	0.008	0.028	0.456	0.152	68.7 %
	0.997		0.018		[0.223 ~ 0.935]		
rs742132	0.999	C	0.227	0.538	1.056	1	7.1 %
	0.989		0.218		[0.887 ~ 1.257]		
rs1183201	0.999	T	0.153	6.39E^−9^	0.572	**1.39E** ^**−7**^	99.8 %
	0.067		0.239		[0.473 ~ 0.692]		
rs12356193	0.999	G	0.002	0.659	1.43	1	7.2 %
	0.997		0.002		[0.288 ~ 7.1]		
rs17300741	0.999	G	0.063	0.133	1.27	0.581	19.2 %
	0.997		0.050		[0.928 ~ 1.736]		
rs505802	0.999	A	0.208	9.88E^−4^	0.747	**0.007**	72.2 %
	0.997		0.260		[0.628 ~ 0.889]		

Results with p value less than 0.05 are shown in boldface

^a^calculated by FDR

^b^the allele that the reported OR correlates with

Genotype and allele frequency distributions in case–control cohorts were shown in Table 3. Three SNPs, rs780094 in *GCKR*, rs1183201 in *17A1* and rs505802 in *SLC22A12* were significantly associated with gout in our samples. The p values after FDR correction for rs780094, rs1183201 and rs505802 were 1.78E^−4^, 1.39E^−7^ and 0.007, respectively. Each effect allele (the one that the reported OR correlates with) of these three SNPs had a same protective effect on gout with the ORs reported as 0.723, 0.572 and 0.747, respectively. However, the remaining SNPs in *PDZK1, SLC2A9, LRRC16A, SLC16A9* and *SLC22A11* were not replicated in this study. Notably, the logistic regression which considered each of the potential confounding covariates at a time did not change the genetic associations substantially except for uric acid concentrations, indicating that gout was the major contributor to the associations independent of such clinical and biochemical characteristics (shown in Table 4). The power values for these SNPs ranged from 7.1 % to 99.8 %. Unfortunately, SNP-SNP interactions for these three significant SNPs were not detected (rs780094 vs rs1183201, p = 0.402; rs780094 vs rs505802, p = 0.434; rs1183201 vs rs505802, p = 0.143).

**Table 4:** Sequential logistic regression analyses that considered each of the potential confounding covariates at a time

SNP	age	BMI	SBP	DBP	Glu	Tri	Cho	UN	Cre	UA
rs12129861	0.788	1	1	0.891	1	1	1	1	0.851	1
rs780094	4.46E^−4^	5.47E^−5^	2.36E^−4^	2.03E^−4^	2.97E^−4^	0.025	1.78E^−4^	1.66E^−4^	1.36E^−4^	1
rs734553	0.094	0.183	0.182	0.2	0.19	0.116	0.178	0.167	0.167	0.19
rs742132	1	1	1	1	1	1	1	1	1	1
rs1183201	9.52E^−9^	2.05E^−8^	4.69E^−8^	9.80E^−8^	2.85E^−8^	1.48E^−6^	5.49E^−8^	5.17E^−8^	3.5E^−8^	0.005
rs12356193	1	1	1	1	1	1	1	1	1	1
rs17300741	0.712	0.831	0.575	0.426	0.52	0.238	0.648	0.544	0.766	0.19
rs505802	0.004	0.01	0.005	0.007	0.003	0.013	0.006	0.006	0.005	0.779

All p values above were estimated by FDR at the same time

### Association of eight SNPs with phenotype details and biochemical indicators

Both SUA levels and primary gout are complicated traits and diseases, being closely correlated with several other metabolic factors [39]. We checked all participants’ questionnaires and confirmed that all cases were first diagnosed with gout on our gout clinic without receiving any relevant medication. Therefore, we decided to conduct this genotype–phenotype analysis in all participants to assess the association of these SNPs with collected clinical characteristics. We found significant association of uric acid concentrations with three gout-related SNPs (rs780094 in *GCKR*, corrected p = 3.94E^−5^; rs1183201 in *SLC17A1*, corrected p = 0.005; rs505802 in *SLC22A12*, corrected p = 0.003) and of triglycerides with rs780094 (located in *GCKR*, corrected p = 2.96E^−4^). The remaining SNPs showed no significant associations with all collected clinical data (shown in Table 5). The results provide further evidence indicating that gout is the major contributor to the associations.

**Table 5:** Associations between clinical data and genotypes of the eight SNPs

	rs12129861	rs780094	rs734553	rs742132	rs1183201	rs12356193	rs17300741	rs505802
Age	0.615	0.615	0.963	1	0.963	0.615	1	1
BMI	1	1	1	1	1	1	1	1
SBP	1	0.301	1	0.334	1	1	1	1
DBP	1	1	1	1	1	1	1	1
Glu	1	1	1	1	1	1	1	1
Tri	1	**2.96E** ^**−4**^	1	1	0.131	1	1	1
Cho	1	1	1	1	1	1	1	1
UN	0.8	1	1	0.8	1	1	0.315	0.8
Cre	1	1	1	1	1	1	1	1
UA	0.117	**3.94E** ^**−5**^	1	1	**0.005**	1	1	**0.003**

All p values were estimated by FDR at the same time. Results with p value less than 0.05 are shown in boldface

## Discussion

This study was performed to assess the association of eight SUA correlated SNPs first identified in Europeans with phenotype gout in Han Chinese males. Three SNPs, rs780094 in *GCKR,* rs1183201 in *SLC17A1* and rs505802 in *SLC22A12* were confirmed to be significantly associated with gout and SUA concentrations. Notably, the logistic regression which considered each of the potential confounding covariates at a time did not change the genetic associations substantially except for SUA concentrations, indicating that gout was the major contributor to the associations independent of such clinical and biochemical characteristics. While the remaining SNPs were not replicated. We also found a significant association of triglycerides with rs780094 in *GCKR*.

**Fig. 1:**
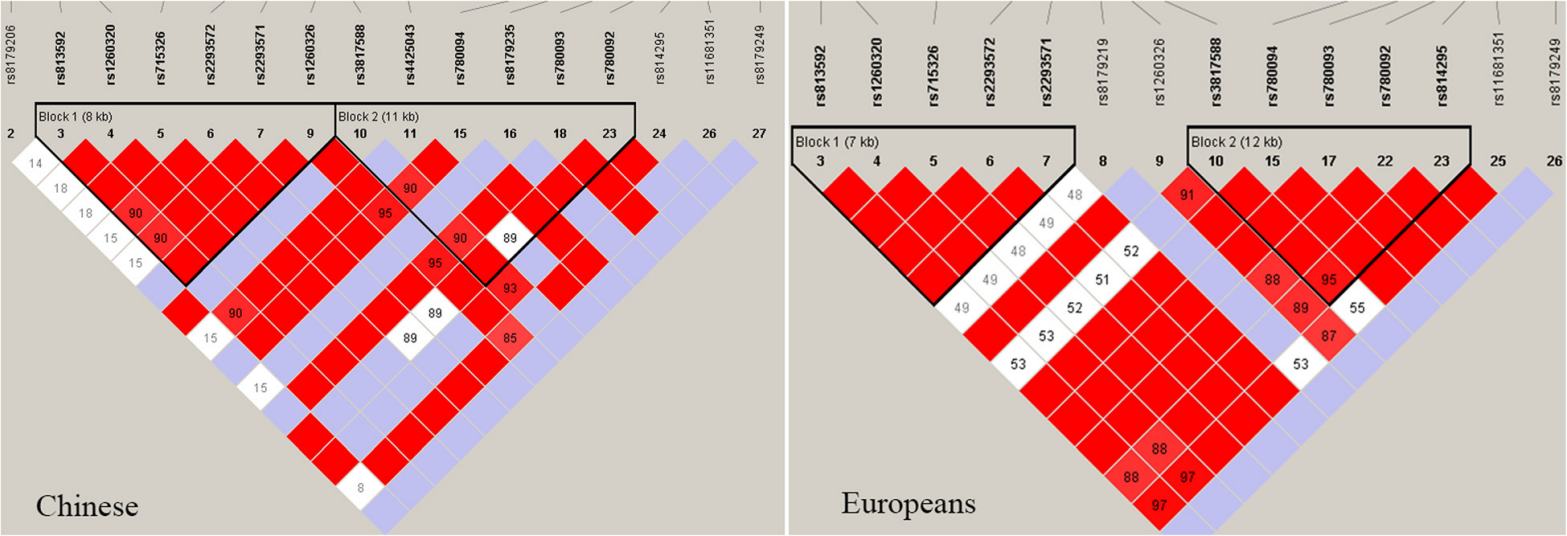
LD plot for Chinese and Europeans, respectively

SNP rs780094 in the *GCKR* gene was found to have a strong association with gout in our samples, of which both *p* value and effect direction were consistent with our previous study by Wang J et al. [23]. The data sets of the two studies were not overlapped, demonstrating its true effect on gout risk in Chinese Han males. However, this association was not replicated in Germany [20], whereas validated in Japan [22] and New Zealand [24]. The direction of the association was consistent among these populations; however, we note that the minor allele frequency (MAF) between the Asians and Europeans was different: the minor allele for Chinese and Japanese was ‘G’ with MAF of 0.473 and 0.444 respectively, consistent with HapMap data (MAF = 0.366 and 0.401, respectively); while the minor allele for Germany and New Zealand population was ‘A’ with MAF of 0.400 and 0.344 respectively, consistent with HapMap-CEU data (MAF = 0.394). Besides, we extracted the genotype data across the gene for Han Chinese and Europeans from the HapMap database and explored the difference of genomic structure and LD between Chinese and Europeans using HaploView software (version 4.2). LD plot for the two populations is shown in Fig. 1. SNP rs780093 in the *GCKR* gene was in very high LD with rs780094 (r^2^ = 1.0) in both Chinese and Europeans. However, the linkage degree around rs780094 in Europeans was obviously higher than that of Chinese. Therefore, the differences of genetic background in different populations indeed exist and more validation tests with larger sample size are needed across various populations. The *GCKR* gene encodes the glucokinase regulatory protein, which regulates glucokinase (GCK) activity [40]. GCK, also known as hexokinase 4, catalyzes the phosphorylation of glucose into glucose-6-phosphate and thus modulates hepatic glucose homeostasis [41]. ‘A’ allele of rs780094 has been observed to be associated with lower fasting glucose level and higher triglycerides in different ethnic groups [42–45]. In this study, ‘A’ allele was also observed to be associated with higher triglycerides in Han Chinese individuals, but not with glucose level or other biochemical indicators. A study from Hong Kong reported that the interaction between rs1799884 and rs780094 weakened the correlation of rs780094 with fasting glucose level in Chinese population [46]. Rs780094 AA genotype frequency in Chinese Han population is much higher compared with European populations (27 % and 14 %, respectively) and thus the interaction between SNPs in Han Chinese is stronger. Therefore, it’s much harder to observe the association of rs780094 with fasting glucose in Chinese population, which urgently needs to be confirmed in larger samples. Although the association of rs780094 with gout has been indeed corroborated in Han Chinese, Japanese and New Zealand populations, the pathway of this locus on gout development is still completely unknown. In the future, relevant functional experiments are needed to delineate the exact involvement of this gene in gout.

The next significant locus in this study was rs505802 within the *SLC22A12* gene. The *SLC22A12* locus encodes urate anion exchanger 1 (URAT1), an organic anion transporter (OAT) involved in the non-voltage-dependent exchange of uric acid for organic anions [47]. Many studies have shown significant associations between SNPs in *SLC22A12* and SUA levels or/and gout. For example, two independent GWAS of SUA level for Croatia population and African Americans identified rs505802 and rs12800450 to be significant loci respectively [48, 49]. Another GWAS by Kamatani et al. and a meta-analysis by Okada et al. both from Japan discovered rs506338 and rs504195 to be associated with SUA concentrations respectively[13, 14]. Follow-up replications across multiple ethnics including New Zealand populations, Japanese and Han Chinese successively identified additional SNPs in *SLC22A12* gene associated with gout or hyperuricemia [22, 24, 27]. SNP rs3825018 (in complete LD with rs505802) also achieved nominal significance in New Zealand case–control sample sets (p = 0.002) [50]. However, this association was not confirmed in German population [20]. And we noted that the MAF of this SNP in Chinese and Germans was different: the minor allele for Chinese was ‘A’ with MAF of 0.260, consistent with HapMap-CHB (MAF = 0.244); while the minor allele for Germans was ‘G’ with MAF of 0.315, consistent with HapMap-CEU (MAF = 0.274) [20]. The different genotype distributions across multiple ethnics may at least partly account for the discordant results.

The strongest association in the present study was rs1183201 within *SLC17A1* gene. *SLC17A1* encodes the solute carrier family 17 member 1, also known as sodium phosphate transport protein 1 (NPT1), which has been identified as a urate transport protein [51]. GWAS or meta-analysis of SUA level among different populations has shown inconsistent results for *SLC17A1* loci: rs1183201 was confirmed to be associated with SUA level among Japanese [13] and Europeans [8, 17] rather than Chinese [11] and Croatia population [48]. Two independent replication studies provided strong evidence for a role of rs1183201 in gout in New Zealand Caucasians [24, 25] and rs1165196 (in high LD with rs1183201, r^2^ = 0.915 in HapMap-JPT) was also replicated in Japanese population [22]. However, it was not associated with gout in a recent study by Wan W et al. [35]. The inconsistent results may indicate the complex pathogenesis of gout, which needs more studies to better elaborate the etiology for such different presentations.

However, we did not detect the association with gout for the remaining SNPs. It’s noteworthy that these negative SNPs could only explain less than 0.20℅ of variability on SUA level except for rs734553 in *SLC2A9* gene with explained variability of 3.53℅ [20]. From the clinical observation, less than ten percent of hyperuricemia patients we examined experienced gouty arthritis in their lifetime. Uric acid metabolism is a risk factor, but cannot fully explain the development of gouty arthritis [1]. We speculate that there may be a different pathogenesis between the two conditions, which may explain our negative results at these SUA sensitive loci. In the power analysis, we note that the power of rs12129861, rs742132 and rs12356193 was as low as 11.4 %, 7.1 % and 7.2 % respectively, almost insufficient to detect their association with gout in such a sample size. Considering the low power values of these SNPs, the negative results of these loci lacked enough evidence. The results may be false negative due to the small sample size, which urgently needs to be confirmed in the further study. In addition, rs734553 was significantly associated with gout in Germans [20], however, the association was not replicated in the present study. This might result from the very low MAF of 0.018 in Chinese males compared to that of 0.253 in Germans.

Almost all kinds of biological traits and complex diseases are results of multiple gene interaction and thus understanding gene interactions on disease is indeed necessary. However, in the present study, SNP-SNP interactions were not detected for the three significant SNPs. In contrast, there was a weak epistasis between rs734553 and rs742132 in the study by Stark *et al.*, suggesting that there may be differences of gene interactions on gout risk among different populations [20].

Our study had several limitations. We did not evaluate lifestyle factors such as high-purine diet, smoking and drinking, which also had a significant impact on complex diseases including gout. There were two rare variants in our samples, rs734553 and rs12356193, a finding consistent with the HAPMAP database. Traditional association method lacks sufficient power to detect such rare variants and second-generation sequencing is more valuable. Several genes including *SLC2A9* possess obvious gender differences on gout risk [9] and understanding the genetic mechanisms of female gout is equally important. But due to less than 5 % of our outpatients were female, not sufficient to conduct a statistical analysis, this study focused on Han Chinese males only.

## Conclusions

Three SUA correlated SNPs, rs780094 in *GCKR*, rs734553 in *SLC2A9* and rs505802 in *SLC22A12* were confirmed to be associated with gout arthritis in Han Chinese males. Considering genetic differences among populations and complicated pathogenesis of gout arthritis, more validating tests in independent populations and relevant functional experiments are suggested in future.

## Availability of Supporting Data

The supporting data underlying the results was included as additional files.

## Abbreviations

SUA: Serum uric acid
SNPs: Single nucleotide polymorphisms
GWAS: Genome-wide association studies
LD: Linkage disequilibrium
HWE: Hardy-Weinberg equilibrium
ORs: Odds ratios
CI: Confidence intervals
FDR: False Discovery Rate
BMI: Body mass index
GCK: Glucokinase
MAF: Minor Allele Frequency
URAT1: Urate anion exchanger 1
OAT: Organic anion transporter
NPT1: Sodium phosphate transport protein 1
