# Erratum to: Polymorphisms in *GCKR*, *SLC17A1* and *SLC22A12* were associated with phenotype gout in Han Chinese males: a case–control study

**DOI:** 10.1186/s12881-015-0237-3

**Published:** 2015-10-28

**Authors:** Zhao-Wei Zhou, Ling-Ling Cui, Lin Han, Can Wang, Zhi-Jian Song, Jia-Wei Shen, Zhi-Qiang Li, Jian-Hua Chen, Zu-Jia Wen, Xiao-Min Wang, Yong-Yong Shi, Chang-Gui Li

**Affiliations:** Shandong Gout Clinical Medical Center, The Affiliated Hospital of Qingdao University, 16 Jiangsu Road, Qingdao, 266003 China; Shandong Provincial Key Laboratory of Metabolic Disease, The Affiliated Hospital of Qingdao University, 16 Jiangsu Road, Qingdao, 266003 China; Bio-X Institutes, Key Laboratory for the Genetics of Developmental and Neuropsychiatric Disorders (Ministry of Education), Shanghai Jiao Tong University, Shanghai, 200030 China

## Erratum

Unfortunately the original version of this article [1] contained a mistake. It came to the authors’ attention that the corresponding authors of the article were not listed correctly, an error which arose during production. Both Yong-Yong Shi (yongyongshi@gmail.com) and Chang-Gui Li (licgqd@163.com) should have been listed as joint corresponding authors.

The full author list should read:

Zhao-Wei Zhou^1,2^, Ling-Ling Cui^1,2^, Lin Han^1,2^, Can Wang^1,2^, Zhi-Jian Song^3^, Jia-Wei Shen^3^, Zhi-Qiang Li^3^, Jian-Hua Chen^3^, Zu-Jia Wen^3^, Xiao-Min Wang^1,2^, Yong-Yong Shi^3*^and Chang-Gui Li^1,2*^

*Corresponding authors: Chang-Gui Li licgqd@163.com and Yong-Yong Shi yongyongshi@gmail.com.
